# Specific Expression of a New Bruton Tyrosine Kinase Isoform (p65BTK) in the Glioblastoma Gemistocytic Histotype

**DOI:** 10.3389/fnmol.2019.00002

**Published:** 2019-01-24

**Authors:** Luca Sala, Giovanni Cirillo, Gabriele Riva, Gabriele Romano, Carlo Giussani, Annamaria Cialdella, Antonio Todisco, Assunta Virtuoso, Maria Grazia Cerrito, Angela Bentivegna, Emanuela Grassilli, Antonio Ardizzoia, Emanuela Bonoldi, Roberto Giovannoni, Michele Papa, Marialuisa Lavitrano

**Affiliations:** ^1^School of Medicine and Surgery, University of Milano-Bicocca, Monza, Italy; ^2^Laboratory of Neuronal Networks, University of Campania “Luigi Vanvitelli”, Naples, Italy; ^3^Unit of Neurosurgery, School of Medicine and Surgery, Neuroscience Center, University of Milano-Bicocca, San Gerardo Hospital, Monza, Italy; ^4^Department of Oncology, ASST Lecco, Lecco, Italy; ^5^Department of Pathology, ASST Lecco, Lecco, Italy

**Keywords:** Bruton’s tyrosine kinase, p65BTK, gemistocytes, glioblastoma, biomarker

## Abstract

Bruton’s tyrosine-kinase (BTK) is a non-receptor tyrosine kinase recently associated with glioma tumorigenesis and a novel prognostic marker for poor survival in patients with glioma. The p65BTK is a novel BTK isoform involved in different pathways of drug resistance of solid tumors, thus we aimed to investigate the expression and the putative role of p65BTK in tumors of the central nervous system (CNS). We selected a large cohort of patients with glial tumors (*n* = 71) and analyzed the expression of p65BTK in different histotypes and correlation with clinical parameters. Sections were stained with glial fibrillary acidic protein (GFAP), p53, epidermal growth factor receptor (EGFR), S100, vimentin, and epithelial membrane antigen (EMA) antibodies. Glioma stem cell (GSC) lines, isolated from glioblastoma multiforme (GBM), were treated with different concentrations of ibrutinib, a specific inhibitor of BTK, in order to evaluate their metabolic activity, mitotic index and mortality. Moreover, an orthotopic xenotransplant of GSC from human GBM was used to evaluate the expression of p65BTK in the brain of immunodeficient mice. p65BTK was expressed in GSC and in gemistocytes in human gliomas at different histological grade. We found a significant correlation between BTK expression and low-grade (LG) tumors (*p* ≤ 0.05) and overall survival (OS) of patients with grade III gliomas (*p* ≤ 0.05), suggestive of worst prognosis. Interestingly, the expression of p65BTK remained restricted exclusively to gemistocytic cells in the xenograft mouse model. Ibrutinib administration significantly reduced metabolic activity and mitotic index and increased mortality in GSC, highlighting the specific role of p65BTK in cell proliferation and survival. In conclusion, our data demonstrated that p65BTK is expressed in glioma tumors, restricted to gemistocytic cells, has a key role in GSC and has a bad prognostic value, thus highlighting the importance of future research for targeted therapy of human gliomas.

## Introduction

Protein tyrosine kinases (PTKs) play an important role in the control of many cellular processes and signaling, including cell migration, metabolism, survival, cell proliferation and differentiation (Endicott et al., 2012). Therefore, perturbation of PTKs signaling results in dysregulated kinase activity and has been implicated in malignant transformation. Accordingly, several tyrosine kinase inhibitors are widely used as a treatment for an increasing number of tumors (Noble et al., 2004; Lamoral-Theys et al., 2012; Gocek et al., 2014).

Bruton’s tyrosine-kinase (BTK) is a non-receptor tyrosine kinase (Mohamed et al., 2009), belonging to the Tec family of kinases (Smith et al., 2001), expressed in all cell lineages of the hematopoietic system, except for T cells. BTK is a 77KD protein essential for B-lymphocyte development, differentiation and signaling (Satterthwaite et al., 1998) and is currently involved in both physiological and oncogenic pathways through B-cell receptor regulation (Seda and Mraz, 2015). Recently, high expression of BTK was associated with glioma tumorigenesis (Wei et al., 2016) and found to be a novel prognostic marker for poor survival in patients with glioma (Yue et al., 2017). The BTK inhibitor ibrutinib is a promising new therapeutic strategy for glioma, blocking the proliferation, migration and invasion properties and inducing apoptosis and autophagy of glioma cells, targeting the Akt/mTOR pathway (Wang et al., 2017).

The p65BTK is a novel BTK isoform transcribed from a different promoter than p77BTK and post-transcriptionally regulated by the mitogen-activated protein kinase (MAPK) pathway (Grassilli et al., 2016), involved in drug resistance of some solid tumors (colorectal, breast, ovarian and non-small-cell lung cancer; De Luca et al., 2012; Lavitrano et al., 2013). We previously showed that p65BTK is a potent oncogene acting downstream of RAS and its targeting by ibrutinib has an anti-proliferative effect on CRC cell lines (Grassilli et al., 2016). These results suggest that p65BTK could represent an ideal target for new advanced drugs: p65BTK targeted inhibition was reported in ovarian (Conconi et al., 2017) and colon cancer, restoring the apoptotic response to chemotherapy (Ianzano et al., 2016). These observations prompted the investigation of the expression and the putative role of p65BTK in tumors of the central nervous system (CNS).

Glioblastoma multiforme (GBM) is the most aggressive high-grade (HG) brain astrocytoma (grade IV, according to the World Health Organization—WHO—classification), and characterized by brief clinical history, rapid progression and poor overall survival (OS; Urbańska et al., 2014). Among low-grade (LG) astrocytomas, the grade II gemistocytic astrocytoma (GemA) is a frequent precursor of GBM and is characterized by glial fibrillary acidic protein (GFAP) expressing cells, called gemistocytes, that show a typical angular shape with plump, glassy, eosinophilic cell body (Avninder et al., 2006), voluminous homogeneous cytoplasm, eccentric nucleus, numerous nucleoli (Krouwer et al., 1991) and long, branching processes. A cut off of 20% gemistocytes of the whole tumor cells is required for the histopathologic diagnosis of GemA, according to the Krouwer’s guidelines (Avninder et al., 2006). Early p53 mutation was reported (Watanabe et al., 1998) and clinical experience has provided evidence of an aggressive behavior, a rapid progression, increased malignant transformation, and reduced OS compared to other cytotypes (38 months compared to 82 months for LG fibrillary and protoplasmic astrocytomas; Babu et al., 2013), although the biology of gemistocytes is not currently understood.

Due to the growing interest in new potential biomarkers for diagnosis, prognosis, and new advanced therapies, we analyzed the expression of p65BTK in different histotypes of human glial tumors and correlation with clinical parameters. To explore the role of p65BTk in gliomas, we cultured glioma stem cell (GSC) lines from patients affected by glioblastoma and evaluated their metabolic activity, mitotic index and mortality after exposure to ibrutinib, a selective potent BTK inhibitor. Finally, an orthotopic xenotransplant of GSC from human GBM in mice was used to confirm the selectivity of p65BTK expression in the gemistocyte cell population.

## Materials and Methods

### Patients

We selected a group of 71 patients affected by glial tumors afferent to the division of Oncology in the Lecco Hospital from 2006 to 2015. We progressively collected clinical and baseline data concerning age, gender, WHO grade, histopathology, OS (excluding patients affected by ependymoma tumors) calculated from the date of the surgery to the exitus. Among these patients, we selected a subgroup of 32 patients, recording data concerning surgery, chemo/radiotherapy, OS, and progression free survival (PFS), calculated from the surgery to the evidence of disease progression.

### Tissue Samples Processing, Immunochemistry and Immunohistochemistry

All samples used in the present study are leftover tissues of deceased patients belonging to the Pathology section of the Lecco Hospital. All patients provided written informed consent for experimental research on their leftover tissues according to the Italian authority laws for genetic data (G.U. n. 8 15/12/2016), and privacy (G.U. n. 9 15/12/2016) and in compliance with the General Data Protection Regulation (EU Directive 2016/679). The protocol was approved by the local Ethics Committee of the Lecco Hospital. Tissue samples were anonymized, included in paraffin, cut with a rotative microtome (Leica RM2245, Leica Microsystems Srl—Italy) at 3 μm, then mounted on polarized glass (72 Superfrost Plus, Kaltek, Padova, Italy), and incubated overnight at 37°C. Sections were then transferred at 72°C in the Bond Dewax Solution for paraffin removal. The unmasking procedure was performed by incubating sections with the Bond Epitope Retrieval Solution (ER1 or ER2) or with an enzymatic solution (ES) according to the specific antibody. The antigen-antibody reaction was revealed by the Bond Polymer Refine Detection kit (DS9800, Leica Microsystems Srl—Italy). Sections were blocked at room temperature (RT) in 10% normal serum in 0.01 M PBS/0.25% Triton-X100 for 1 h. Sections were incubated with primary reagent/antibodies for 15 min (short protocol) or 30 min (long protocol), washed several times in PBS and incubated with the specific secondary antibody for 8 min (short protocol) or 15 min (long protocol), then with the polymer for 8 min (short protocol) or 15 min (long protocol) at RT. The reaction product was imaged by 3,3′-diaminobenzidine tetrahydrochloride (DAB; Mixed DAB Refine) for 3 min, followed by a wash in PBS. The sections were counterstained with hematoxylin for 30 s. Two expert neuropathologists evaluated the staining intensity and percentage of positive tumor cells as negative (−), weak (1+), moderate (2+) or strongly positive (3+).

We selected five tissue samples of different grade gliomas (not included in the patients’ group) and we produced a tissue microarray (TMA) to standardize the immunohistochemistry technique using the specific antibody for p65BTK (BN30).

### Antibodies: Clone, Protocol, Unmasking Treatment and Dilution

The following antiserum has been used for manual immunohistochemistry: polyclonal rabbit antisera directed against anti-p65BTK BN30 (1:2,500, heat mediated antigen retrieval in Sodium Citrate 10 mM, pH 6.0). The following antibodies were used for automated immunohistochemistry: monoclonal antibody directed against Ki-67 (clone K-2, long protocol, ER2, 1:300, Leica), monoclonal/antibody directed against TP53 (clone D0-7, short protocol, ER1, 1:150, Leica), monoclonal antibody directed against GFAP (clone GA-5, short protocol, ER2, 1:75, Leica), monoclonal antibody directed against epidermal growth factor receptor (EGFR; clone EGFR.2, long protocol, ER2, 1:25, Leica), polyclonal antibody directed against S100 (rabbit antisera, short protocol, ES, 1:1,000, Leica), monoclonal antibody directed against Vimentin (clone V9, short protocol, ER2, 1:200, Leica), monoclonal antibody directed against epithelial membrane antigen (EMA; clone GP1.4, short protocol, ER2, 1:100, Leica), monoclonal antibody directed against mitochondria (clone 133-1, long protocol, ER1, 1:2,000, Biogenex).

BTK+, when moderate (2+) or strong (3+) positivity with BN30 staining; BTK−, when negative (−) o weak (1+) positivity with BN30 staining. Positivity for p53 when >10% positive cells with anti TP53 clone D0-7.

### Glioma Cell Lines and Cell Cultures

The GSC lines (GBM2, G144, G166, GBM04) were isolated from patients affected by glioblastoma and characterized for their stemness properties from a genetic and molecular point of view (Baronchelli et al., 2013). All the GSC lines were already expanded *in vitro* as stable cell lines and used as powerful model for studying their biology and testing drug susceptibility, furthermore their cytogenomic and epigenomic profiles were well characterized (Riva et al., 2014, 2018; Cilibrasi et al., 2017). GSC were cultured in adherent culture condition using 10 mg/ml laminin (Invitrogen) in a proliferation permissive medium composed by DMEM F-12 and Neurobasal 1:1 (Invitrogen), B-27 supplement without vitamin A (Invitrogen), 2 mM L-glutamine, 10 ng/ml recombinant human bFGF and 20 ng/ml recombinant human EGF (Miltenyi Biotec), 20 UI/ml penicillin and 20 g/ml streptomycin (Euroclone; complete medium).

### Drug and Treatments

Ibrutinib (Selleckchem, Houston, TX, USA) was dissolved in dimethylsulfoxide (DMSO) to make a 100 mM stock solution, then diluted to the final selected concentrations (0.1–1–10–20 μM) and stored in aliquots at −80°C. Dissolved in DMSO had no effect on cell survival [evaluated by 3-(4,5-dimethylthiazol-2-yl)-2,5-diphenyl tetrazolium bromide (MTT) assay]. Cell culture treatments were assessed following administration of ibrutinib at different concentrations for 24, 48 and 72 h.

### MTT Assay

Cell metabolic activity was assessed by MTT assay (Sigma-Aldrich, Germany), as already described (Cilibrasi et al., 2017). Cells were seeded in 96 well-plates at a density of 4 × 104 cells/well in 100 μl of culture medium and incubated at 37°C. After 24 h, ibrutinib at various concentrations (0.1, 1, 10 and 20 μM) was added to cell culture medium. After the drug incubation time (24, 48 or 72 h) MTT solution (1 mg/ml, Sigma) was added to each well and cells were incubated for 3 h at 37°C. Therefore, formazan was solubilized in absolute ethanol and the absorbance of the dye was measured spectrophotometrically with FLUOstar Omega microplate reader (BMG Labtech) at 595 nm. The percentage of inhibition was determined by comparing the absorbance values of drug-treated cells with that of un-treated controls: [(treated-cell absorbance/untreated cell absorbance) × 100]. The results reported are the mean values of three different experiments performed at least in triplicate.

### Mitotic Index Analysis

The mitotic index was assessed in order to evaluate the effect of ibrutinib on cell proliferation. 2 × 10^6^ cells were seeded in T-25 cm^3^ in 5 ml of medium. Subsequently, cells in exponential growth phase were treated with 20 μM ibrutinib for 48 h. Then metaphase chromosome spreads were obtained using standard procedures (Riva et al., 2014; Cilibrasi et al., 2017).

The chromosomes were QFQ-banded using quinacrine mustard (Roche) and slides were mounted in Mc Ilvaine buffer. Slides were analyzed using Nikon Eclipse 80i fluorescence microscope (Nikon) equipped with a COHU High Performance CCD camera. Mitotic index was evaluated counting the percentage of mitosis, scoring at least 1,000 nuclei. Data were obtained as mean values derived from two independent experiments.

### Trypan Blue Dye Exclusion Assay

Cells were plated in 60 mm Petri dishes at a density of 1.2 × 10^6^ cells/dish and cultured overnight. Then, cells were treated with ibrutinib 1 and 20 μM for 48 and 72 h and stained using the trypan blue dye (Sigma-Aldrich, Germany) to count dead cells. The treated samples were compared with the untreated controls. The results reported are the mean values of four different experiments.

### Western Blotting

Twenty micrograms of each protein extract were then separated using NuPAGEBis-Tris pre-casted mini gel 10% (Invitrogen), blotted using iBlot system (Invitrogen) on Nitrocellulose membranes and incubated with the rabbit polyclonal anti-p65BTK (BN49, 1:2,500; Grassilli et al., 2016), and mouse monoclonal anti-p77BTK (Becton Dickinson, 1:500), primary antibodies.

After incubation with appropriate secondary antibodies, Super Signal West Dura Extended duration substrate (Thermo Scientific) was added to the membranes and chemiluminescent signal was digitally acquired by G:BOXXT4 (Syngene). Samples from spleen were used as a control of p77BTK expression. Blots were then re-probed with anti-actin as a loading control.

### Orthotopic Xenograft of GSCs in a Mouse Model

The consortium of mouse models of human tumors (MHCC) proposed specific criteria for the ideal tumor model. The MHCC recommendations suggest that animal models of brain tumors might be supervised by a qualified and expert neuropathologist and the histology classified according to the WHO scheme. All the experiments involving animals described in the present manuscript were carried out in accordance with national and European Guidelines for use and care of laboratory animals (EU Directive 2010/63), according to a protocol specifically approved by the Italian Ministry of Health (Approval N. 268/2012-B).

Immunodeficient male NOD/SCID mice (*n* = 8) of 4–6 weeks were allowed free access to food and water and maintained under a 12/12 h light/dark cycle in pathogen-free iron sheet cages. Mice were anesthetized with an intraperitoneal (IP) injection of ketamine and xylazine (118 mg/kg and 1.8 mg/kg, respectively). Surgery (20 min for each mouse) was performed by an expert neurosurgeon, under a laminar flow hood in sterile conditions. An ophthalmic gel was used during the surgery. G144 GSCs were treated with trypsin 0.05% (Euroclone), counted and diluted in a suspension of sterile PBS. Before the procedure, the cellular vitality was verified by trypan blue. A skin incision was made over the skull and a small opening through the cranial bone using a small drill (1 mm rostral to Bregma, 2 mm laterally, width 2.5 mm). The cellular solution was mixed and 5 μL of the solution, containing 2 × 10^5^ G144 cells, loaded in a Hamilton micro-syringe and slowly injected in the striatum of each NOD/SCID mouse.

Control sham animals (*n* = 3) were injected with a sterile saline solution. The injection needle was left *in situ* for 2–3 min before being removed. The small opening was then filled with bone wax and the skin sutured.

Animals were monitored and weighed daily. Mice were anesthetized with isoflurane and sacrificed by decapitation. All NOD/SCID mice were sacrificed after 6 months or earlier in case of signs of distress, the brain removed and processed.

### Mouse Tissue-Sections Preparation

These procedures were performed by the Neuropathology unit of the Desio Hospital. Brains from NOD/SCID mice were immersed in a 4% formaldehyde solution for 24 h and then in a 70% ethanol solution. Samples were treated through graded alcohols, xylene and liquid paraffin on a pre-set time scale. Coronal sections 5 μm thick were cut with a sliding microtome then mounted onto chrome-alume gelatine coated slides. Every 50 μm, a section was hematoxylin-eosin stained.

### Immunohistochemistry on Xenograft’s Tissues

Paraffin sections were xylene treated (two changes, 5 min each) and then gradually rehydrated through graded alcohols scale. Slides were then washed in PBS for 15 min and treated for the antigen retrieval procedure, in a solution of sodium citrate 10 mM at 95°C for 10 min, followed by 20 min at RT, and finally washed in PBS for 10 min.

The activity of endogenous peroxidase was silenced by H_2_O_2_ immersion at 3% for 10 min. Slides were treated using the blocking solution (ImmPRESS Reagent Kit, Vector, DBA ITALIA, Segrate, Italy). Sections were incubated for 1 h with the primary antibody against GFAP (1:400, Sigma Aldrich, Milan—Italy); or anti-p65BTK BN30 (1:2,500) diluted in PBS 1X/Tween 20 0.5% and then washed in PBS and incubated with the HRP-secondary conjugated antibody (ImmPRESS Reagent Kit, Vector, DBA ITALIA, Segrate, Italy). The reaction was revealed by DAB (Sigma, Milan—Italy) 0.5 mg/ml in Tris-HCl for 7 min. Sections were washed in distilled water, counterstained with a Nissl solution (cresol violet 0.1% for 8 min at 37°C), washed again with distilled water and dehydrated with ethanol solutions (95 and 100% for 5 min), cleaned with xylene for 3 min. Finally, slides were coverslipped, imaged and analyzed by Aperio ScanScope (Leica Microsystems Srl–Italy).

### Statistical Analysis

Statistical analysis was carried out performing Yates’ chi-square test (mitotic index) or *t*-test (MTT assay, Trypan blue exclusion assay, Western blot), Wilcoxon-Mann-Whitney test for continuous variables (age, Ki67, PFS, OS), Fisher’s exact test for categorical or dichotomous variables (IHC markers, EGFR, p53, tumor grade). OS and PFS curves were obtained with Kaplan-Meier method. The Log-rank test was used to compare Kaplan-Meier curves. *P* value was set at ≤ 0.05. Raw data were exported and converted using SigmaPlot 10.0 with SigmaStat 3.5 integration (SPSS Erkrath, Germany) in frequency distribution histograms.

## Results

### Analysis of p65BTK Expression in Gliomas

The correlation of BTK expression with glioma tumorigenesis (Wei et al., 2016) and with poor survival in glioma patients (Yue et al., 2017), and the discovery of a novel oncogenic BTK isoform (p65BTK) in solid tumors (Lavitrano et al., 2013; Grassilli et al., 2016), prompted the investigation of the p65BTK expression in gliomas.

**Figure 1:**
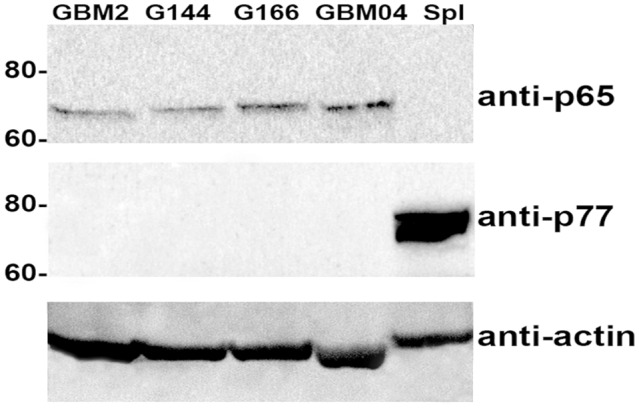
p65BTK expression in glioma stem cell (GSC) lines (GBM2, G144, G166 and GBM04) protein extracts. Spleen protein extract was used as a control for the expression of Bruton’s tyrosine-kinase (BTK) known isoform (p77BTK). Western blots were probed with BN49 antibody for p65BTK expression, and with a commercial anti-BTK antibody (BD Transduction Laboratories) for the expression of p77BTK.

To this extent, protein extracts of human GBM cell lines were analyzed by western blotting and immunolabeled with a p65BTK specific antibody (BN49). The BN49 antibody detected a 65 KDa specific band in GBM cells (Figure 1), suggesting that the BTK expressed in these cancer cells is the oncogenic isoform previously described by our group in colon cancer (Grassilli et al., 2016).

We then selected five cases of gliomas of different histological grade that were analyzed with a single staining, by means of TMA on the cerebral tissues.

### p65BTK Expression in Different Glioma Histotypes

In Table 1, we show clinical and baseline data of all the patient included in the study (*n* = 71): 45 cases of GBM (grade IV), 6 cases of oligoastrocytomas (OA), 3 cases of anaplastic oligodendrogliomas (aOD), 1 case of anaplastic astrocytoma (aA; grade III = 10 cases), 3 cases of GemA, 2 cases of OD, 2 cases of OA (grade II = 16 cases). We also selected 9 cases of ependymomas (Ep).

**Table 1:** Clinical-demographic data of patients.

	All patients	BTK+	BTK−
**Patients** (*n*)	71	13	58
**Age** (mean ± SD)	58.76 ± 14.82	62.46 ± 10.80	57.93 ± 15.54
**Gender** *n* (% of patients)	M = 37 (52.11)	M = 9 (69.23)	M = 28 (48.28)
	F = 34 (47.89)	F = 4 (30.77)	F = 30 (51.72)
**Histotype (Grade WHO)**			
*n* (% of patients)			
GBM (IV)	45 (63.38)	5 (38.46)	40 (68.98)
aOA (III)	6 (8.45)	1 (7.69)	5 (8.62)
aOD (III)	3 (4.22)	2 (15.39)	1 (1.72)
aA (III)	1 (1.41)	1 (7.69)	0
GemA (II)	3 (4.22)	3 (23.08)	0
OA (II)	2 (2.82)	0	2 (3.44)
OD (II)	2 (2.82)	1 (7.69)	1 (1.72)
Ep (II)	9 (12.68)	0	9 (15.52)

*Abbreviations: WHO, World Health Organization; GBM, glioblastoma multiforme; aOA, anaplastic oligoastrocytoma; aOD, anaplastic oligodendroglioma; aA, anaplastic astrocytoma; GemA, gemistocytic astrocytoma; OA, oligoastrocytomas; OD, oligodendroglioma; Ep, ependymoma; SD, standard deviation; M, male; F, female*.

**Figure 2:**
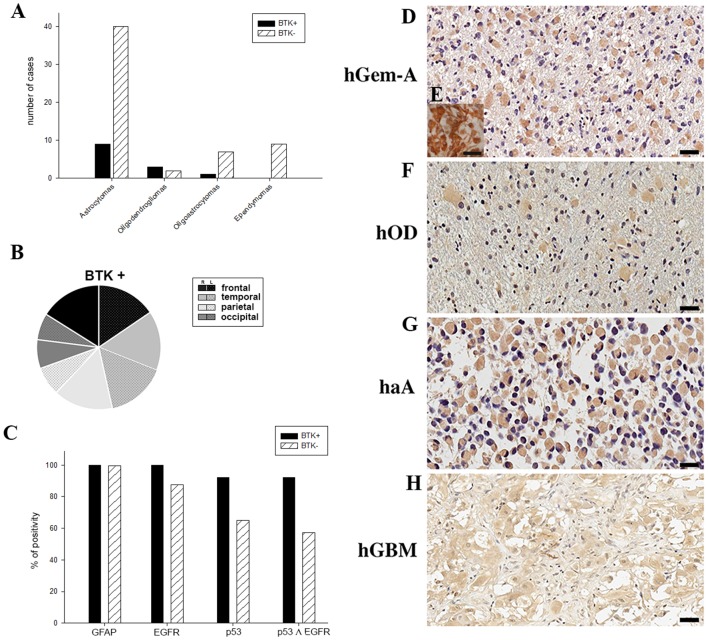
**(A)** p65BTK expression in patients with different gliomas. **(B)** Anatomical location and side of BTK+ tumors. **(C)** Positivity percentage of glial fibrillary acidic protein (GFAP), epidermal growth factor receptor (EGFR), p53 and p53-EGFR co-expression in BTK+ and BTK−. **(D,F–H)** BTK expression in gemistocytes in human gliomas of different grade (hGemA, human gemistocytic astrocytoma; hOD, human oligodendroglioma; haA, human anaplastic astrocytoma; hGBM, human glioblastoma multiforme; magnification 40×, scale bar: 20 μm). **(E)** GFAP positive gemistocytes in hGemA (magnification 60×, scale bar: 25 μm).

The expression of p65BTK was then tested on formalin-fixed paraffin-embedded (FFPE) samples from all 71 patients by immunohistochemistry using BN30 specific antibody. BTK+/BTK− in relation to histotype subgroups are showed in Table 1. More in detail, p65BTK positivity was found in samples from 13 patients (BTK+), among these, nine were astrocytomas, 3 OD and 1 OA and were distributed in the brain lobes as follows: 4 in the frontal lobe (31%, 2 left, 2 right), 4 in temporal lobe (31%, 2 left, 2 right), 3 in the parietal lobe (23%, 2 right, 1 left) and 2 in occipital lobe (15%, 1 right, 1 left; Figures 2A,B). No significant correlation was found between p65BTK expression and anatomical location of the tumor. BN30 staining in all nine samples of Ep was negative.

The analysis of BTK expression in the whole group of low grade (II WHO) and high grade (III-IV WHO) gliomas demonstrated that p65BTK (BTK+) is significantly more expressed in low grade (II WHO) gliomas (*p* ≤ 0.05). No significant correlation was found between age, gender and BTK+.

All samples were also characterized analyzing a panel of markers commonly used in clinics. The IHC analysis and the percentage of staining positivity for GFAP, EGFR, p53, Ki67, S100, EMA and vimentin, is reported in Table 2.

**Table 2:** Clinical data and immunohistochemical positivity of clinical markers in gliomas.

Histotype	Case/Patients *n*	Mean age ± SD	Male gender %	EGFR+ %	GFAP+ %	p53+ %	Ki67+ %	S100+ %	EMA+ %	Vimentin+ %
GBM	45	64.44 ± 11.6	55.56	88.89	100	64.4	28	/	/	/
aOA	6	45.5 ± 15.53	16.67	83.33	100	100	25	/	/	/
aOD	3	61.67 ± 3.51	66.67	100	100	100	34	/	/	/
aA	1	53	100	100	100	100	10	/	/	/
GemA	3	58.67 ± 3.79	66	100	100	66.67	8.5	/	/	/
OA	2	41.5 ± 14.85	50	100	100	100	32.5	/	/	/
OD	2	37.5 ± 3.54	50	100	100	50	17.5	/	/	/
Ep	9	47.44 ± 18.54	44.4	/	100	/	4	100	66.67	100

*Abbreviations: EGFR, epidermal growth factor receptor; GFAP, glial fibrillary acidic protein; EMA, epithelial membrane antigen; GBM, glioblastoma multiforme; aOA, anaplastic oligoastrocytoma; aOD, anaplastic oligodendroglioma; aA, anaplastic astrocytoma; GemA, gemistocytic astrocytoma; OA, oligoastrocytomas; OD, oligodendroglioma; Ep, ependymoma; SD, standard deviation; /, no positive staining*.

All BTK+ samples revealed positive reactivity for GFAP and EGFR, 92.31% were also positive for p53 (Figure 2C). The mean proliferation index revealed by Ki67 reactivity was 21.58% on all BTK+. BTK negative (BTK−) patients were all positive for GFAP, as expected; 87.76% were positive for p53 (Figure 2C) and the mean proliferation index was 28.33%. Co-expression of EGFR and p53 was found in 92.3% of BTK+ patients and in 57.14% BTK− patients (Figure 2C).

### p65BTK Expression Is Restricted to Gemistocytic Cells

The p65BTK is expressed exclusively in gemistocytic cells and this specificity does not depend on grade and histotypes. The expression of p65BTK is selective for the cytoplasm and present in all gemistocytic cells in all the examined samples. We observed a specific and selective immunoreactivity to p65BTK for gemistocytic cells across patients with different histological WHO grade (Figures 2D–H), in particular in the three cases of “pure” GemA (grade II WHO; Figure 2D). All gemistocytes were p65BTK and GFAP positive cells (Figure 2E). As expected, p65BTK was not expressed in any of the nine cases of Ep, demonstrating and confirming the specificity for the gemistocytic cytotype, neither in a control tumor (two samples of oncocytomas), in which the p65BTK staining was negative.

### Correlation Between p65BTK Expression and Clinical History

For the OS and PFS analysis, p65BTK+ vs. p65BTK− patients were compared, independently from the WHO grade. The mean OS was 16.5 months, with a median of 16 months (interval 10–24) in BTK+ patients, while 22.44 months, with a median of 15 months (interval 8–69) in BTK− patients.

The median PFS was 6.5 months for BTK+ patients and 6 months for BTK− patients. The mean PFS was 7.67 months with a median PFS of 6.5 months in BTK+ patients, while 11.09 months with a median PFS of 6 months (interval 10–24) in BTK−.

No statistically significant correlation was pointed out between BTK+ and BTK− patients according to OS (*p* = 0.67) and PFS (*p* = 0.48).

**Figure 3:**
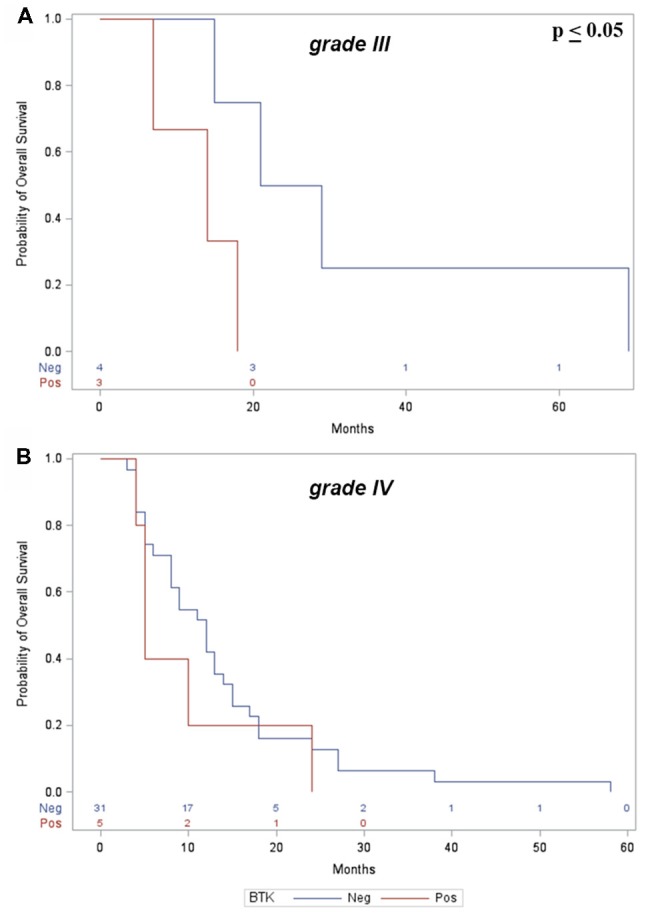
**(A)** Kaplan-Meier curves show significant correlation between p65BTK+ and OS in grade III gliomas. **(B)** No significant correlation was found with grade IV gliomas.

However, a significant correlation between OS and BTK+ was found in the subgroup analysis of patients with grade III gliomas (*p* ≤ 0.05; Figure 3A); in patients with grade IV gliomas, BTK+ also correlates with worst prognosis although without significant correlation (*p* = 0.36; Figure 3B).

### p65BTK Expression in a Murine Model of Glioblastoma Xenotransplants

**Figure 4:**
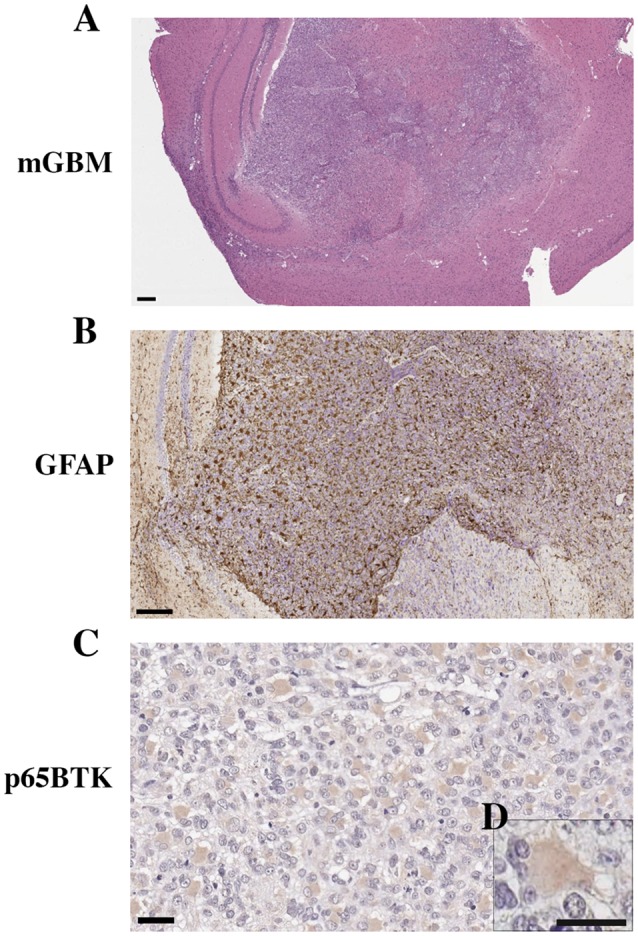
Orthotropic xenotransplant of hGBM (G144 cells) in mouse. **(A)** Gross anatomy of GBM in the mouse brain (magnification 2.5×, scale bar: 400 μm). **(B)** GFAP expression in the mouse GBM (magnification 10×, scale bar: 200 μm). **(C)** BTK expression in gemistocytes of xenografted human GBM tumors (magnification 40×, scale bar: 20 μm). **(D)** Higher magnification of gemistocytes (magnification 60×, scale bar: 10 μm).

We generated a murine model of human GBM (Figure 4A) to evaluate the expression of p65BTK in cell proliferation in an *in vivo* setting and in particular to verify the selective localization of p65BTK in the gemistocytic cells. Human GSC G144 line injected into the mouse brain developed a GBM with the same histotype and oncogenicity of the primary tumor. As observed in human samples, the neoplastic tissue expressed the specific marker of astrocytic differentiation GFAP (Figure 4B). Moreover, p65BTK expression was specific and selective for gemistocytic cells also in the neoplastic tissue isolated from GBM xenograft stained with BN30 antibody (Figures 4C,D). On the whole, these results indicated that p65BTK expression was maintained in GBM cells from *in vitro* GSC cultures to their growth developing GBM tumor *in vivo*, in which it is restricted to gemistocytic cells.

### Metabolic Activity in Glioma Stem Cells After p65BTk Inhibition

**Figure 5:**
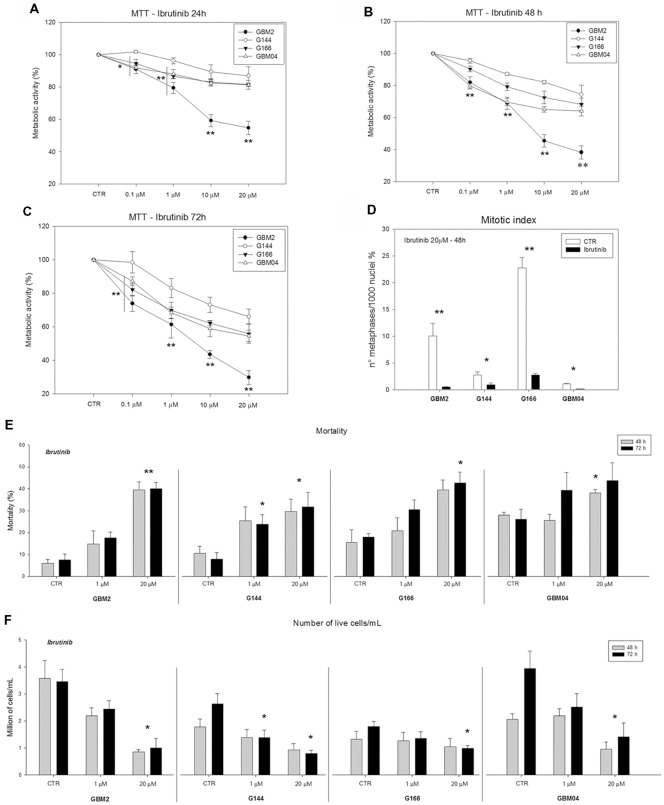
Metabolic, proliferative activity and mortality of GSC lines after exposure to ibrutinib. **(A–C)** Dose- and time-dependent significant reduction of the metabolic activity (3-(4,5-dimethylthiazol-2-yl)-2,5-diphenyl tetrazolium bromide, MTT assay) after exposure to ibrutinib for 24, 48 or 72 h (*p ≤ 0.05; **p ≤ 0.001, treatment vs. control). **(D)** Reduction of mitotic index after treatment with 20 μM ibrutinib for 48 h (*p ≤ 0.05; **p ≤ 0.001, treatment vs. control). **(E)** Mortality essay and **(F)** number of live cells after exposure to ibrutinib 1 or 20 μM for 48 or 72 h (*p ≤ 0.05; **p ≤ 0.001, treatment vs. control).

The effect of ibrutinib, a clinically approved BTK inhibitor, on GSC metabolic activity was determined by MTT assay (Figures 5A–C). Metabolic activity was significantly reduced in GBM2, G144, G166 and GBM04 lines after 24, 48, or 72 h of treatment in a dose-dependent and time-dependent manner, compared to the matching untreated cells (*p ≤ 0.05; **p ≤ 0.001). The most consistent reductions of metabolic activity were obtained after 72 h of treatment with 20 μM ibrutinib: in particular, GBM2 showed a drastic reduction of the metabolic activity (70.2%), GBM04 and G166 displayed a value around 50%, while G144 was the most resistant cell lines (33.8% of reduction). These data suggested that p65BTK inhibition by ibrutinib specifically reduced the metabolic activity of all GSC lines, suggesting a role of p65BTK in cell growth and proliferation.

### p65BTk Inhibition Affect GSC Proliferation

In order to confirm the role of p65BTK in cell proliferation, we treated four human GSC lines with 20 μM ibrutinib and we evaluated the mitotic index after 48 h. Our results indicated that ibrutinib induced a dramatic and significant decrease of mitotic index in all the cell lines compared to the control non-treated cells (*p ≤ 0.05; **p ≤ 0.001; Figure 5D). In conclusion, GSC proliferation was significantly reduced after p65BTK inhibition by ibrutinib treatment.

### p65BTk Inhibition Induces GSC Mortality and Reduced Cell Growth

GSC mortality and cell growth were evaluated by Trypan blue dye exclusion assay. Inhibition of p65BTK by administration of ibrutinib for 48 and 72 h, induced a significant increase of dead cell percentage and an important decrease of live cell numbers, in a dose-dependent way, in all the cell lines analyzed compared to the corresponding untreated cells (Figures 5E,F). After 72 h of 20 μM ibrutinib exposure, the mortality was about 40% for GBM2, GBM04 and G166, while was 31.7% for G144. In the same conditions, the live-cell number of the ibrutinib treated samples were a third of the control ones in GBM2, GBM04 and G144. These data confirmed the role of p65BTK in cell proliferation and indicated that ibrutinib had a dose-dependent cytotoxic and growth-inhibitory effect.

## Discussion

In the recent years, research on tumor biology is progressively conditioning the diagnostic and therapeutic approach, leading to a personalized medicine and a customized treatment (Jameson and Longo, 2015). In the field of oncology, the identification of new theranostic targets will lead to the development of new drugs with the potential of overcoming the current therapeutic approach and allowing more effective treatments.

This study emerges from the evidence that p65BTK, the new isoform of the BTK, is a powerful oncogene and is expressed in different solid tumors (Lavitrano et al., 2013). Both p65BTK levels and its oncogenicity depend on the RAS/ERK/MAPK pathway and its inhibition primarily affects colon cancer cells with mutated TP53, suggesting that p65BTK could be a new therapeutic target in the colon cancer, in which both deregulation of the RAS/ERK/MAPK pathway and loss of p53 function have been shown (Grassilli et al., 2016).

Gliomas are the most frequent primary brain tumors in the adult population and GBM is the most frequent glioma (2–3 new cases per year per 100,000), with the worst prognosis (survival at 5 years <5%). Despite the different clinical trials carried out in the recent years, little has changed in the treatment of this disease, hence the need to investigate new markers and new potential therapeutics.

Deregulations of the RAS/ERK/MAPK pathway are also found in some gliomas, mainly in secondary glioblastomas, in which mutations of p53 are very frequent. The p53 mutation is a distinctive sign of the gemistocytic cytotype, that characterizes the WHO grade II GemA but is found in other gliomas with a different degree and histotype (Hede et al., 2011). Gemistocytes are GFAP positive cells (Watanabe et al., 1998), precisely recognized for their morphological features by the pathologists. The presence of at least 20% of gemistocytes in a glial neoplasia clearly defines the GemA histotype, which is responsible for a marked trend in the progression and therefore it is a negative prognostic factor (Krouwer et al., 1991). The reason for this more aggressive behavior is not completely understood: gemistocytes, intriguingly, show a low proliferative capacity and resemble some extent to the reactive astrocytes, reported in several pathological conditions.

In this study, we selected different histotype of gliomas with a different grade that, although not extensive, typify different primary glial neoplasms. We initially studied 71 patients, for 62 of them we reconstructed the OS data and for 32 patients the complete clinical history from the onset of the disease, including treatments and recurrences. This allowed us to study the correlations between the clinical and therapeutic parameters and the immunophenotype properties of neoplastic tissues.

The p65BTK was expressed in 13 positive cases of different glial tumor histotypes, including GBM, aA, aOD and aOA, GemA and OD except for the Ep, independently of the grade. Only few cells showed p65BTK expression, with a constant positive correlation between the occurrence of the gemistocytes and the expression of p65BTK. In contrast, we found an intense staining for p65BTK in the three cases of pure gemistocytic phenotypes.

Our orthotopic mouse model of human GBM, obtained injecting GSCs in immunodeficient mice, confirmed the specificity of p65BTK expression in gemistocytes.

Exposure of GSC lines to ibrutinib, a potent inhibitor of BTK, significantly reduced metabolic activity and mitotic index and increased cell mortality. These data highlight the role of p65BTK in GSC proliferation and survival, thus consolidating its putative role for targeted therapies.

Although there is no correlation between the expression of p65BTK and the degree of disease, there is a significant difference between WHO low grade (II) and high grade (III-IV) tumors (*p* ≤ 0.05). Gemistocytes are more frequently expressed in LG tumors and progressively decrease at disease progression. BTK+ cases have a greater p53 expression compared with BTK–patients and the co-expression of EGFR and p53 is significant in BTK+ patients (*p* ≤ 0.05), suggesting that gemistocytes are present in the lower stages of the disease, where the p53 mutation is characteristic.

Clinical data showed no correlation between BTK expression, gender and age of the patients and anatomical location of the tumors. Analysis of OS, however, showed a significant correlation between p65BTK expression and grade III gliomas, as showed in Figure 3A, indicative of worst prognosis.

In conclusion, IHC of p65BTK in glial tumors could be helpful in defining prognosis and in identifying a possible therapeutic target. Further studies are necessary to confirm the signal specificity with molecular techniques and the negative prognostic value of the p65BTK expression in the different grades of gliomas. Moreover, we aim to test the role of p65BTK in brain tumorigenesis and development, by transplanting G144 cells in mice brains before and after treatment with p65BTK inhibitors.

## Author Contributions

LS, GC, GRo, GRi, AC, AT, AV, MC, EG and CG: analysis and interpretation of the data. GC, AB, AA, EB, RG, MP, ML and CG: experimental design, analysis and interpretation of the data.

## Conflict of Interest Statement

The authors declare that the research was conducted in the absence of any commercial or financial relationships that could be construed as a potential conflict of interest.

## Abbreviations

aA: anaplastic astrocytoma
GBM: glioblastoma multiforme
GSC: glioma stem cells
GemA: gemistocytic astrocytoma
IHC: immunohistochemistry
OA: oligoastrocytomas
aOA: anaplastic oligoastrocytomas
OD: oligodendrogliomas
aOD: anaplastic oligodendrogliomas
OS: overall survival
PFS: progression free survival
WHO: World Health Organization.
